# RETRACTION: Repressor Element 1 Silencing Transcription Factor (REST) Governs Microglia‐Like BV2 Cell Migration via Progranulin (PGRN)

**DOI:** 10.1155/np/9896268

**Published:** 2026-05-12

**Authors:** Neural Plasticity

RETRACTION: T. Yu, Y. Lin, Y. Xu, et al., “Repressor Element 1 Silencing Transcription Factor (REST) Governs Microglia‐Like BV2 Cell Migration via Progranulin (PGRN),” *Neural Plasticity*, 2020, 8855822, https://doi.org/10.1155/2020/8855822.

The above article, published online on 25 November 2020 in Wiley Online Library (wileyonlinelibrary.com), has been retracted by agreement by the Journal Editor‐in‐Chief, Michel Baudry; and John Wiley & Sons Ltd. The retraction has been agreed due to errors in the figures as follows:

• The graph in Figure 2b is identical to the graph in Figure 2b of [1] by some of the same authors, despite the western blots appearing different.
• The Figure 2c REST bands for 0 µM and 1 µM are vertically and horizontally rotated versions of the REST bands in Figure 3a of [1].
• The graph in Figure 4a is identical to graph in Figure 3b of [1], despite the western blots appearing different.
• Figure 4e is identical to the 5µM Aß panels in Figure 3d of [1].

Following these concerns being raised, the authors were unable to provide the raw data for the study and therefore requested the retraction of the article. The article is therefore retracted with the agreement of the Editorial Board and the authors.
